# The two‐fold cost of sex: Experimental evidence from a natural system

**DOI:** 10.1002/evl3.1

**Published:** 2017-05-03

**Authors:** Amanda K. Gibson, Lynda F. Delph, Curtis M. Lively

**Affiliations:** ^1^ Department of Biology Indiana University Bloomington Indiana 47405; ^2^ Department of Biology Emory University Atlanta Georgia 30322

**Keywords:** All‐else‐equal assumption, asexual reproduction, evolution of sex, experimental evolution, paradox of sex, *Potamopyrgus antipodarum*, sexual reproduction, two‐fold cost of males

## Abstract

Over four decades ago, John Maynard Smith showed that a mutation causing asexual reproduction should rapidly spread in a dioecious sexual population. His reasoning was that the per‐capita birth rate of an asexual population would exceed that of a sexual population, because asexual females do not invest in sons. Hence, there is a cost of sexual reproduction that Maynard Smith called the “cost of males.” Assuming all else is otherwise equal among sexual and asexual females, the cost is expected to be two‐fold in outcrossing populations with separate sexes and equal sex ratios. Maynard Smith's model led to one of the most interesting questions in evolutionary biology: why is there sex? There are, however, no direct estimates of the proposed cost of sex. Here, we measured the increase in frequency of asexual snails in natural, mixed population of sexual and asexual snails in large outdoor mesocosms. We then extended Maynard Smith's model to predict the change in frequency of asexuals for any cost of sex and for any initial frequency of asexuals. Consistent with the “all‐else equal” assumption, we found that the increase in frequency of asexual snails closely matched that predicted under a two‐fold cost.

**Impact Summary**

A rare asexual mutant arises in an otherwise sexual population. This asexual female need not mate with a male to produce sons and daughters. Instead, she simply clones herself, producing asexual daughters. Evolutionary theory predicts that this asexual lineage will spread rapidly through the population, driving the sexual lineages rapidly extinct. The reason is that sexual females must spend ∼50% of their resources making sons, which cannot themselves make offspring. The growth rate of the sexual population is thus predicted to be half that of the asexual population. This cost is called the “two‐fold cost of males.” Yet sex abounds in nature. Since the development of this theory, evolutionary biologists have sought advantages for sex that could explain its paradoxical persistence. In this study, we take a step back and ask: do sexuals actually pay a two‐fold cost? Though the cost of sex is a critical assumption of the paradox of sex, there are no direct estimates of the cost. To estimate the cost of sex, we conducted an experiment using snails collected from a natural population where sexual and asexual individuals coexist. The snails were reared in large, outdoor mesocosms, and the experiment was replicated in four separate years. We found that the asexual snails increased in frequency in all four years. We then extended a previous model on the two‐fold cost so that we could estimate the cost of sex based upon our experimental data. We found that the observed increase in asexual frequency matched that predicted for a two‐fold cost of sex. Our results are thus consistent with theoretical predictions. Hence, for sex to be maintained in natural populations, there must be strong selection favoring sexual over asexual reproduction.

## Introduction

The cost of males (Maynard Smith 1971, 1978), along with Williams’ “cost of meiosis” (1971, 1975), sparked an enduring paradox in evolutionary biology: sexual reproduction is costly but is maintained in most eukaryotic species. In his original model, Maynard Smith (1971, 1978) assumed that sexual females invest 50% of their resources into sons, while asexual females invest 100% of their resources into clonal daughters. He also assumed that sexual and asexual females are equally fecund and that the survivorship of their offspring is equal (“all‐else‐equal” assumption). Under these conditions, the model predicts that the per‐capita birth rate of an asexual population would be twice that of a sexual population (two‐fold cost). An asexual mutant should therefore double in frequency when rare and rapidly replace the sexual population. Sex, however, abounds. This inconsistency between theoretical expectation and nature instigated the ongoing hunt for forces that counterbalance the short‐term costs of sex.

Though the cost of sex is the foundation of the paradox of sex, the costs are vastly understudied relative to its benefits. Without a cost of sex, there is no need to test hypotheses for the maintenance of sex. For example, intrinsic differences between sexual and asexual females can violate the all‐else‐equal assumption (e.g., costs of elevated ploidy in asexuals), reducing the cost of sex predicted by theory (Lehtonen et al. 2012; Meirmans et al. 2012). Alternately, intrinsic differences may augment the cost of sex (e.g., energetic costs associated with mating), in which case a stronger selective advantage for sex would be required to explain coexistence.

There are no direct experimental measures of the cost of sex. Several empirical studies have addressed the critical all‐else‐equal assumption of Maynard Smith's model, with mixed support. Asexual and sexual females have similar fecundity and/or offspring survival in only five of the ten cases reviewed in Meirmans et al. (2012) (e.g., snails—Jokela et al. (1997b); Crummett and Wayne (2009); rotifers—Stelzer (2011)). In the other cases, the transition to asexuality is accompanied by reduced fecundity or survival, violating the all‐else‐equal assumption. A few additional studies have shown that the frequency of asexual individuals increases over time in mixed populations, suggesting that sex is indeed costly to some extent (Browne and Halanych 1989; Jokela et al. 1997b; Stelzer 2011). But a crucial question remains: exactly how costly is sex?

Here, we directly estimated the cost of sex in a natural system. To do so, we established seminatural mesocosms using *Potamopyrgus antipodarum* snails collected directly from a natural population in which asexual and sexual females coexist. In the experimental populations, the frequency of asexual snails increased significantly from parent to offspring generation. We then expanded upon Maynard Smith's original model by relaxing his assumption that asexuals are rare. To estimate the net cost of sex in *P. antipodarum*, we fit this simple model to our experimental data. We found that the change in the frequency of asexuals matched that predicted under a two‐fold cost of sex. As such, the net cost of sex in this system is consistent with Maynard Smith's critical “all‐else‐equal” assumption.

## Methods

### NATURAL HISTORY

Obligately sexual lineages of *P. antipodarum* coexist with obligately asexual lineages. Sexual males and females are diploid. Asexual lineages arise by mutation from local sexual genotypes (Neiman et al. 2005) and are primarily triploid females (higher ploidies have been found) (Neiman et al. 2011). Prior studies supported the all‐else‐equal assumption for fecundity in *P. antipodarum*: sexual and asexual females are similar in size at reproductive maturity, brood at similar rates, and have an equal number of eggs per brood (Jokela et al. 1997a; Jokela et al. 1997b; Paczesniak 2012). We conducted the same comparisons for snails in our experimental populations and in the field population from which they were derived (S.I. I). Additional intrinsic fitness differences may exist. Specifically, we did not know whether sexual and asexual females are equally likely to survive to reproduction, or if they produce an equivalent number of viable offspring. We tested this assumption in the present study.

### EXPERIMENTAL TEST

#### Establishing seminatural mesocosms

We established outdoor seminatural mesocosms to experimentally measure the change in frequency of asexual snails. In each of four years, we obtained data from six mesocosms initiated with 800 juvenile snails. Experimental mesocosms were populated each year with field‐collected snails, giving 24 total replicates. By using field collections, we maintained the relative frequencies and genetic diversity of clonal and sexual lineages of the natural population. This is important, as the asexual population of snails consists of many genetically distinct clones (Dybdahl and Lively 1995) and their frequencies change rapidly (Jokela et al. 2009; Paczesniak et al. 2014). Our experimental results therefore reflect the natural variation present in the field.

In January 2012–2015, juvenile *P. antipodarum* were collected by passing a net through *Isoetes kirkii* vegetation (∼1 meter depth) at sites along the southwestern coast of Lake Alexandrina (Fig. S1A). In 2012, the sampled sites were Swamp and Camp. In 2013 and 2014, the sampled sites were 1^st^ Fence, Swamp, 2^nd^ Fence and West Point. In 2015, we substituted Halfway for West Point. These sites have been well‐studied since 1994 (Jokela et al. 1997b), so we knew that large numbers of snails could be found there and that both reproductive modes would be represented. Ecological conditions are similar at these nearby sites. Hosts from these sites are also undifferentiated at neutral loci, consistent with substantial gene flow (Fox et al. 1996; Paczesniak et al. 2014).

We transferred all field samples to the University of Canterbury's Edward Percival Field Station in Kaikoura, NZ and sieved them with a 1.7 mm sieve to obtain juveniles. Body size reflects age, and the 1.7 mm sieve effectively differentiates reproductively mature males and females (>2.5 mm in length) from juveniles. We used juveniles to establish mesocosms, because we aimed to minimize selection by parasites. Juveniles have low rates of infection with sterilizing trematodes (Levri and Lively 1996), the dominant parasites of *P. antipodarum (*Hechinger 2012 *)*. Therefore, using juveniles minimized the proportion of our starting populations that was castrated. Importantly, the few infected snails in the mesocosms were unable to transmit the infection. Trematodes require additional host species (e.g., waterfowl) to complete their life cycle (Hechinger 2012).

In 2013–2015, we counted out and combined 200 snails from each of the four sites to give 800 snails per mesocosm. In 2012, we similarly combined snails from the two sampled sites. Each mesocosm was thus representative of the whole sampled region of the lake. We transferred experimental replicates to 1000 L Dolav box pallets, filled with ∼800 L of water (Fig. S1B). These were located outside the Edward Percival field station in NZ, so experimental populations experienced natural seasonal variation in temperature, weather, and photoperiod. We covered the mesocosms with shade cloth and fed the snails with spirulina for ∼2 weeks. The mesocosms were then left unattended from mid‐February until early January of the next year. We added no additional food during this time. Snails obtained food from algae growing in the tanks and from other environmental inputs. Under natural temperature conditions, a year is sufficient time for juveniles to mature and reproduce, but insufficient time for their offspring to reproduce. Therefore, only two generations were present in the mesocosms at the end of the experimental year. At this point, we emptied the mesocosms and sieved the experimental populations at 1.4 mm to separate parent and offspring snails into discrete generations. This small size cut‐off enabled us to effectively separate parents from offspring, which were very young and thus very small. Occasionally, offspring failed to pass through the sieve and remained in the parental collection. These offspring were easily identified by size and shell morphology.

#### Data collection

After separating parent and offspring snails at the end of an experimental year, we collected a random sample of 150 parents from each mesocosm. These parents were immediately dissected under a microscope to determine shell length, sex, brooding status, brood size, and infection status. Mean infection frequency with sterilizing trematodes was 9.82 ± 0.79% SEM. These individuals were infected as juveniles, prior to field collection. No infections were acquired in the mesocosms because the trematodes’ definitive hosts were absent. Because we aimed to measure intrinsic differences in birth rates between asexual and sexual females, we excluded infected (i.e., castrated) individuals from our analyses. This removed an obvious force that can alter the relative fitness of sexual and asexual females. The heads of all dissected females were individually frozen, shipped to Indiana University (IN, USA), and stored at –80°C. Males were assumed to be sexual diploids (Neiman et al. 2011).

We retained a random sample of the offspring (>200) from each mesocosm. This sample was maintained at the Edward Percival Field Station for ∼5 weeks, with regular water changes and *ad libitum* spirulina feedings. Offspring were then transported alive to Indiana University and promptly frozen at –80°C for storage.

Flow cytometry was conducted as in Gibson et al. (2016). Triploid asexual females can be differentiated from diploid sexual females and males because their ∼50% larger genome size is detected as elevated fluorescence of nuclei. We analyzed 3000 nuclei per sample for parents and 2000 for offspring. For the parental generation, we ran flow cytometry on females only. Parents were sufficiently developed to differentiate males from females, and male snails are exclusively diploid and sexual at Lake Alexandrina (Neiman et al. 2011). For each mesocosm, we analyzed 62.13 ± 4.16 SEM females randomly subsampled from those dissected and frozen. For the offspring generation, we ran flow cytometry on both males and females, because the offspring were too young to sex. For each mesocosm, we analyzed 70.38 ± 1.62 snails randomly subsampled from the frozen offspring. Samples were excluded if there were fewer than 1000 nuclei obtained for a parental snail or fewer than 400 for an offspring snail, if there was no clear peak in fluorescence, or if the peak fell between the gates that designate regions consistent with diploid versus triploid nuclei. We excluded 6.01 ± 1.49% of parents and 5.38 ± 0.913% of offspring.

#### Statistical analysis

We first determined the number of triploid females versus diploid males and females in our subsamples of the parental and offspring generations. For the offspring generation, we obtained these numbers directly from the flow cytometry results because we ran flow cytometry on male and female offspring. For the parental generation, we ran flow cytometry on a subset of females only. We obtained the number of triploid and diploid female parents directly from the flow cytometry results. We used the ratio of male to female snails in the entire mesocosm sample to calculate the number of males consistent with a subsample of females of this size. We then calculated the number of diploid individuals (females + males) (Table S1). Infected individuals were excluded from these calculations to remove differential selection due to parasites.

To determine if the frequency of asexual individuals increased from the parent to offspring generation, we fit a logistic model with the number of triploid (female) and diploid (male and female) individuals in a replicate generation as the binomial response variable (logit link function). Generation (parent, offspring), year (2012–2015), and their interaction were categorical predictor variables. We initially fit this model as a generalized estimating equation (function geeglm in geepack) (R Core Team 2013) to account for autocorrelation of parent and offspring snails derived from the same experimental population (Liang and Zeger 1986; Zeger and Liang 1986; Ziegler and Vens 2010). There was no autocorrelation of generations from the same mesocosm. Therefore, we fit a simpler generalized linear model (function glm in R). We tested the significance of each effect using a likelihood ratio test of models with and without the effect of interest. We calculated the fold‐increase and 95% confidence intervals using the odds ratios for generation from a logistic model that excluded the interaction term, the profile likelihood confidence intervals for the odds ratios, and the mean proportion of asexual individuals in the parental generation. In the Supporting Information (II), we report the results of the logistic model fitted with the quasi‐binomial distribution to account for overdispersion. The results are qualitatively identical to those with the binomial distribution. We therefore report the binomial results in the main text for ease of interpretation.

### MODEL FIT

#### Candidate models

In the Results (“Extended Model”), we used basic population genetic theory to predict the increase in the frequency of asexuals as a function of their initial frequency and the cost of sex. We applied equation (2) of this model (see Results) to our mesocosm data to ask if the observed proportion of asexual offspring (*q_t + 1_*) was consistent with a two‐fold cost of sex (*c* = 2) given the initial (parental) proportion of snails that were asexual in experimental mesocosms (*q_t_*). We formulated four candidate models: (1) no cost of sex (*c* = 1), (2) a twofold cost (*c* = 2), (3) the maximum likelihood estimate (MLE) of the cost, and (4) the MLE of costs that vary with year (Table 1). We proposed model 4 because the composition of clones and/or the accuracy of the experiment may have differed among years. To model yearly variation, we specified the cost of sex (*c*) as a function of the baseline cost of sex (*c_0_*) in 2012 and a deviation term (*d*) indexed by year.

**Table 1:** Results of model inference and selection

Model			logL	Parameters^b^	AIC_c_	ΔAIC_c_	w
**1**	No cost	$q_{t+1}=q_{t}$	−100.78	1 (θ)	203.74	29.01	0.00
	*c = 1*						
**2**	2‐fold	$q_{t+1}=\frac{2q_{t}}{1+q_{t}}$	−86.27	1 (θ)	174.73	0.00	0.72
	*c = 2*						
**3**	Estimate	$q_{t+1}=\frac{cq_{t}}{1+q_{t}\left(c-1\right)}$	−86.07	2 (θ, *c*)	176.71	1.98	0.27
	*c = MLE*						
**4**		$q_{t+1}=\frac{cq_{t}}{1+q_{t}\left(c-1\right)}$	−85.16	4 (θ, *c*_0_, *d*_2_, *d*_3_, *d*_4_)	183.65	8.93	0.01
	By year^a^	2012: $c=c_{0}$					
	c = MLE*year	2013: $c=c_{0}+d_{2}$					
		2014: $c=c_{0}+d_{3}$					
		2015: $c=c_{0}+d_{4}$					

We proposed four candidate models for our experimental data. These four models assume different values of the cost of sex: (1) no cost (*c=1*); (2) a two‐fold cost (*c=2*); (3) the maximum likelihood estimate (MLE) of the cost; and (4) the MLE of costs that vary with year. Each model is represented in the form of equation (2). We ranked models according to ΔAIC_c_ and evaluated the weight of evidence for each model using *w*, the Akaike weight.

^a^For model 4, the cost was indexed by experimental year. Maximum likelihood estimates of *d_j_* that significantly deviate from 0 indicate that the cost of sex in experimental year *j* differed from that estimated in 2012 (S.I. V).

^b^Total number of estimated parameters. To fit models to experimental data, we assumed a beta‐binomial distribution for the likelihood functions and thus estimated an additional overdispersion parameter θ.

#### Likelihood function

Our four candidate models specified different probabilities of observing the number of triploid offspring in the total number of offspring analyzed, given the proportion of individuals that were triploid in the parental generation. Offspring numbers were obtained directly from the flow cytometry subsample. The proportions of parents that were triploid were obtained from the flow cytometry subsample and the estimated number of males, as described above (Table S1). We assumed a beta‐binomial distribution (R package emdbook, Bolker 2008) for our likelihood function (S.I. III). We used the mle2 function (package bbmle, R) to find maximum likelihood estimates of the parameters. We then obtained the likelihood of each model given our experimental data (24 paired estimates of proportion asexual in parental and offspring generations, *q_t_* and *q_t+1_* respectively).

#### Model comparison

We compared models using Akaike's information criterion (Akaike 1973), corrected for small sample sizes (Sugiura 1978; Hurvich and Tsai 1991) (AIC_c_) and ΔAIC, the difference in AIC_c_ of the focal model and the best model (lowest AIC_c_). (Burnham and Anderson 1998). Roughly, models with ΔAIC values below two have substantial support, models with ΔAIC from 4 to 7 have considerably less support, and models with ΔAIC above 10 have no support (Burnham and Anderson 1998). Because these cut‐offs are only rules of thumb, Burnham and Anderson (1998) (pp. 128–129) advise calculating the ΔAIC value that delineates a 95% confidence set of models. We followed their recommended procedure by bootstrapping our data set 10,000 times with replacement. For each bootstrapped dataset, we fit our four candidate models and calculated ΔAIC for model 2 (best model) by subtracting the minimum AIC value from the AIC value for model 2 in each bootstrap replicate. We identified the value of ΔAIC_2_ that was greater than or equal to 95% of the ΔAIC_2_ values obtained in the bootstrapping analysis. The confidence set of models is defined as those having ΔAIC less than or equal to this limit in the actual data analysis.

We also calculated Akaike weights, *w*, which can be interpreted as the probability that model *i* is the best model among the set of *R* candidate models (Akaike 1978; Burnham and Anderson 1998). Values near 0 indicate that a model is very unlikely to be the best model in the set of candidate models. Lastly, we bootstrapped our data set 10,000 times with replacement and re‐ran model fitting to estimate 95% confidence intervals for parameter estimates.

### ALL‐ELSE‐EQUAL ASSUMPTION

Our estimates of the net cost of sex (*c*) allowed us to test Maynard Smith's original assumption that sexual and asexual females produce the same number of surviving offspring. We calculated the ratio of surviving asexual offspring to surviving sexual offspring (*r*) using our estimates of *c* and the primary sex ratio (*s*) (see “Extended model” in the Results section). We do not know the primary sex ratio of sexual *P. antipodarum* at Lake Alexandrina. Our a priori prediction is a sex ratio of 50% female (*s* = 0.5). The population of *P. antipodarum* is large (Paczesniak et al. 2014), so we predict a Fisherian sex ratio (Hamilton 1967). In addition, related prosobranch snails have chromosomal sex determination with females heterogametic (Barŝiene et al. 2000; Yusa 2007). To estimate a range for the primary sex ratio, we calculated the tertiary sex ratio in our experimental and field populations (i.e., the proportion of females in the adult sexual subpopulations) (Tables S2 and S3). Further details are in the Supporting Information (IV).

## Results

### EXPERIMENTAL TEST

**Figure 1:**
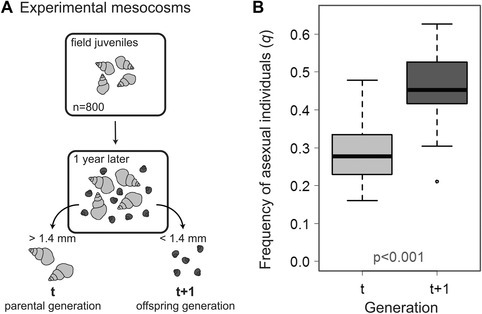
Increase in asexual frequency in experimental mesocosms. (A) Mesocosms were initiated with 800 field‐collected juveniles (gray), which matured to adulthood and produced offspring (black) over the course of one year. Parents (originally juveniles) and offspring were separated by size and split into discrete generations (*t* and *t+1*, respectively). We then estimated the frequency of asexual individuals in parent (*q_t_*) and offspring (*q_t+1_*) generations. (B) The frequency of asexuals increased from the parent (*t*) to offspring (*t+1*) generation. Box plot shows median (black bar), upper, and lower quartiles (limits of box), minimum and maximum (whiskers, excluding outliers), and outliers (dots). The measure of significance is derived from the logistic model reported in the text. Each generation is represented by 24 mesocosms. The numbers of triploid females represented by each mesocosm are: 28.33 ± 1.50 SEM for parents and 23.67 ± 3.60 for offspring for the six mesocosms in 2012; 21.00 ± 1.97 for parents and 37.00 ± 2.29 for offspring in 2013 mesocosms; 16.67 ± 2.75 for parents and 34.33 ± 2.03 for offspring in 2014 mesocosms; and 16.67 ± 1.52 for parents and 27.83 ± 2.82 for offspring in 2015 mesocosms.

We experimentally measured the change in asexual frequency in a single generation of the freshwater snail *P. antipodarum*. Our goal was to directly estimate the net cost of sex in a natural system. To do this, we added juvenile snails sampled from Lake Alexandrina (South Island, New Zealand, Fig. S1A) to six 800‐liter mesocosms (Fig. S1B). The use of field collections ensured the relevance of our results to natural populations. The juveniles matured and reproduced over the course of one year. We then separated parents and offspring by size into discrete generations (*t* and *t + 1*, respectively) to estimate the proportion of asexual individuals in the parents (*q_t_)* and the offspring (*q_t+1_*) generations (Fig. 1A). We replicated the experiment for four years, for a total of 24 independent replicates.

The frequency of asexuals increased 1.60‐fold (95% CI [1.48, 1.73]) from an initial frequency of 29% in the parental generation (Fig. 1B; logistic model, generation: likelihood ratio D = 123.40, df = 1, p < 0.001). There was no variation in the direction of change between years (interaction: D = 2.37, df = 3, p = 0.499), but the overall frequency of asexuals was highest in 2013 and 2014 (odds ratio vs 2012: 2013, 1.70 [1.39, 2.07]; 2014, 1.43 [1.17, 1.75]; 2015, 1.15 [0.94, 1.41]; year: D = 31.90, df = 3, p < 0.001).

The increase in asexual frequency was substantial (1.60‐fold), but clearly less than the two‐fold increase predicted for a two‐fold cost under Maynard Smith's original model. However, asexual snails were not rare at the beginning of the experiment (Fig. 1B), as assumed by Maynard Smith's model. For a two‐fold cost of sex, how much should asexuals increase in frequency when they are not initially rare? In the next section, we answer this question by using basic population genetic theory to predict the increase in frequency of asexuals given any initial frequency and any cost of sex. We then fit this model to our experimental data to estimate the cost of sex for *P. antipodarum*.

### EXTENDED MODEL

We constructed a simple model that relaxes several assumptions of Maynard Smith's model. First, we relaxed the assumption that asexuals are rare. Secondly, we allowed the primary sex ratio of the sexual population to deviate from 0.5. Finally, we relaxed the “all‐else‐equal” assumption that asexual and asexual females produce on average the same number of surviving offspring.

Basic population genetic theory shows that the change in frequency of an allele over one generation is a function of its initial frequency and the strength of selection (Gillespie 1998). We applied this theory to predict the change in asexual frequency as a function of the parental frequency and the net cost of sex. We first write the frequency of asexuals in the next generation (*q_t+1_*) as:

(1) $$q_{t+1}=q_{t}\frac{W_{asex}}{\bar{W}}.$$

where *q_t_* is the initial frequency of asexuals, *W_asex_* is the per‐capita birth rate for asexual females, and $\bar{W}$ is the mean per‐capita birth rate for the mixed population of sexual and asexual individuals: $\bar{W}=q_{t}W_{asex}+\left(1-q_{t}\right)W_{sex}$. Here *W_sex_* is the per‐capita birth rate for the sexual population, which includes males and females.

**Figure 2:**
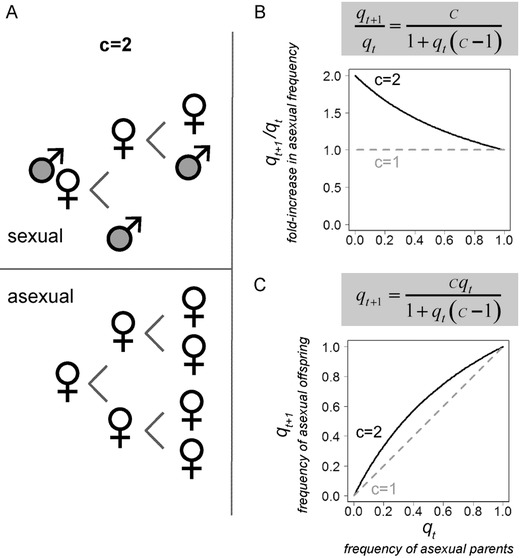
Theoretical predictions for the cost of sex. (A) Under a two‐fold cost of sex (*c* = 2), asexual females can produce twice as many childbearing offspring (females) as sexual females. The net cost *c* is the product of the female fecundity‐survival ratio *r* and the cost of males. Here, sexual and asexual females produce an equivalent number (*n* = 2) of surviving offspring (fecundity‐survival ratio *r* = 1), consistent with the all‐else‐equal assumption. Sexual females make 50% daughters (*s* = 0.5), so the cost of males is two (1/*s* = 2). The total cost of sex is then two (*c = r* * *1/s*). (B) Equation (3) shows the fold‐increase in asexual reproduction: under a two‐fold cost (*c* = 2, black solid line), doubling is observed only at very low starting frequencies of asexual individuals. The proportional increase in asexual frequency declines from two to one as the initial frequency of asexuals (*q_t_*) increases from rarity to fixation. Equation (2)’s corresponding prediction for the frequency of asexual individuals in the offspring generation (*q_t+1_*) is shown in (C). We use equation (2) when fitting models to experimental data. When there is no net cost to sexual reproduction (*c* = 1, gray dashed line), asexuals have no intrinsic birth rate advantage and will not change in frequency.

Let the per‐capita birth rate of the sexual population be a fraction (1/*c*) of the asexual birth rate, where *c* represents the net cost of sex (Fig. 2A), such that Wsex=Wasex/Wasexcc. An estimated value of two for *c* would be consistent with a two‐fold cost of sex, while a value of one would mean that sexual females incur no net cost. By substituting, equation (1) becomes:

(2) $$q_{t+1}=\frac{cq_{t}}{1+q_{t}\left(c-1\right)}.$$

Dividing both sides of equation (2) by *q_t_*, we can calculate the fold‐increase in the frequency of asexuals as:

(3) $$\frac{q_{t+1}}{q_{t}}=\frac{c}{1+q_{t}\left(c-1\right)}.$$

Given a two‐fold cost, equation (3) illustrates that the proportional increase in asexual frequency declines from two to one as the frequency of asexuals (*q_t_*) moves from rarity to fixation (Fig. 2B). For a cost of sex equal to 2 (*c =* 2), a rare asexual mutant will double in frequency, as shown by Maynard Smith. The predicted increase is far less when asexuals are common (e.g., 1.54‐fold for *q_t_* = 30%) (Fig. 2B).

Equation (2) can be rearranged to directly estimate the cost of sex from any starting frequency of asexuals:

(4) $$c=\frac{q_{t+1}\left(1-q_{t}\right)}{q_{t}\left(1-q_{t+1}\right)}.$$

In this model, the parameter *c* represents the net cost of sex, which includes the cost of males weighted by any fecundity‐survival asymmetries in sexual versus asexual females. It is important to deconstruct *c* into its component parts, because the net cost of sex is a function of the cost of males plus many additional factors that may generate asymmetries in the fitness of sexual and asexual females (e.g., costs of mating, costs or benefits associated with ploidy differences). We represent these potential asymmetries using the parameter *r*, the ratio of the mean number of surviving offspring produced by asexual females divided by the mean number produced by sexual females. A value of one for *r* is consistent with the all‐else‐equal assumption: sexual and asexual females produce the same number of surviving offspring. Let the variable *s* be the proportion of resources allocated by sexual mothers to daughters. Assuming that sons and daughters are equally costly (Fisher 1930), *s* represents the proportion of offspring that are daughters in broods of sexual females (i.e., the primary sex ratio). The cost of males is then 1/*s*. The total cost of sex is simply the product of the cost of males and the female fecundity‐survival ratio (Fig. 2A): c=r/rss.

### MODEL FIT

From our experiment in seminatural mesocosms, we observed that the proportional increase in asexual frequency was less than two‐fold. We also observed that asexuals were initially common, not rare. From the basic theory outlined above, we know that, for a two‐fold cost of sex, the proportional increase in asexual frequency is predicted to be less than two‐fold when asexuals are common. Here, we combine our theory and data to ask: is a two‐fold cost the best approximation to our experimental data? Specifically, we used equation (2) (Fig. 2C) to ask if the observed frequency of asexual offspring was consistent with a two‐fold cost of sex given the initial (parental) frequency of asexual snails in experimental mesocosms.

**Figure 3:**
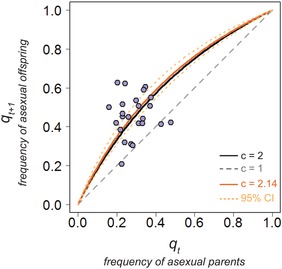
Experimental data are consistent with model predictions of a two‐fold cost of sex. We fit our simple model (Fig. 2C; eq. (2)) to experimental data (Fig. 1B) on the frequency of asexuals *q* in generations *t* and *t+1* in 24 seminatural mesocosms (purple points). We used standard maximum likelihood techniques and Akaike's information criterion to compete different estimates of the cost of sex *c* in *P. antipodarum*. The predicted frequency of asexual offspring (*q_t+1_*) for a given frequency of asexual parents (*q_t_*) is shown for three values of the cost of sex: no cost (*c* = 1, gray dashed line), a twofold cost (c = 2, black solid line), and the maximum likelihood estimate (*c* = 2.14, solid orange line). The 95% confidence intervals of the maximum likelihood estimate include two ([1.81, 2.55], dotted orange lines). Each point represents one mesocosm. For each mesocosm, the average number of triploid parents was 20.67 ± 1.57 SEM and the average number of triploid offspring was 30.71 ± 1.95.

We formulated four candidate models: (1) no cost of sex (*c* = 1), (2) a two‐fold cost (*c* = 2), (3) the maximum likelihood estimate (MLE) of the cost, and (4) the MLE of costs that vary with year. A two‐fold cost of sex (model 2) and the maximum likelihood estimate of the cost (model 3) were the best approximations to our experimental measures of *q_t_* and *q_t+1_* (Table 1). These two models had low ΔAIC and a high weight of evidence in their favor. The likelihood of model 3 was maximized at a cost of sex that slightly exceeds two (*c* = 2.14, 95% CI [1.81, 2.55]), but not significantly so (Fig. 3). We concluded that, given the initial frequency of asexuals in our experimental mesocosms (*q_t_*), the observed frequency of asexuals in the offspring generation (*q_t+1_*) was consistent with a two‐fold cost of sex.

The analysis firmly rejected model 1's assumption of equivalent per capita birth rates of sexual and asexual populations (i.e., no cost of sex). There was also little support for temporal variation in the cost of sex: the ΔAIC for model 4 was relatively large, exceeding the upper limit for our 95% confidence set of models (ΔAIC = 3.94), and the weight of evidence was 0 (Table 1). Parameter estimates also gave little support for yearly variation in cost (S.I. V; Fig. S2).

### ALL‐ELSE‐EQUAL ASSUMPTION

By applying theory to our empirical data, we estimated the net cost of sex *c* to be equal to, or slightly greater than, 2. The net cost of sex *c* is a product of the cost of males (1/*s*; *s* being the primary sex ratio of broods of sexual females) and the ratio of surviving offspring produced by asexual versus sexual females (*r*, the female fecundity‐survival ratio). Under the all‐else‐equal assumption of Maynard Smith's original model, asexual and sexual female make an equivalent number of surviving offspring (*r* = 1). There are many reasons to expect *r* to deviate from 1, such as the energetic costs associated with outcrossing. We were able to test the all‐else‐equal assumption for *P. antipodarum* using our estimates of the net cost of sex *c* and the sex ratio *s* (S.I. IV).

For our *a priori* prediction of *s* equal to 0.5, our estimate of the fecundity‐survival ratio *r* is 1 for model 2 (*c* = 2) and 1.07 (95% CI [0.91, 1.28]) for model 3, consistent with the all‐else‐equal assumption. We also calculated *r* assuming that the primary sex ratio is equal to the tertiary sex ratio calculated from parents in our experimental mesocosms: *s* = 0.61 (S.I. IV). Our estimate of *r* is then 1.22 for model 2 and 1.31 [1.10, 1.56] for model 3. Estimates of *r* above 1 are consistent with our observation that, in the mesocosms, the average brood of an asexual female contained 21% more embryos than that of a sexual female (S.I. I). We conclude that asexual females produce at least as many (i.e., *r =* 1), if not more (e.g., *r =* 1.31), surviving offspring than sexual females. Clearly, a net reduction in fitness does not accompany the transition to asexuality and elevated ploidy in *P. antipodarum*. In fact, this analysis and our life‐history comparisons (S.I. I) suggest that sexual females may pay additional fitness costs beyond just the cost of males.

## Discussion

Here, we provide a direct estimate of the net cost of sexual reproduction in a mixed population of freshwater snails. First, we conducted an experiment in semi‐natural mesocosms (Fig. 1A) to show that asexual snails increase substantially in frequency (1.6‐fold) from parent to offspring generation (Fig. 1B). However, this increase in asexual frequency is less than the two‐fold increase predicted for the two‐fold cost of males under Maynard Smith's original model. We resolve this apparent inconsistency between theory and data by using a standard population genetic approach. The results show that a two‐fold cost of sex manifests as a two‐fold increase in asexual frequency only when asexuals are very rare. When asexuals are common, as in our experiment, a two‐fold cost manifests as a smaller increase in asexual frequency (Fig. 2B). We then applied this model to our experimental data. We found that, given the initial frequency of asexual snails in our mesocosms, the observed frequency of asexual offspring is consistent with that predicted under a two‐fold cost of sex (Fig. 3, Table 1). We conclude that asexual *P. antipodarum* produce at least as many viable offspring as sexual females, resulting in at least a two‐fold fitness cost for sexual reproduction. Our estimate of the net cost of sex in *P. antipodarum* is thus consistent with Maynard Smith's two‐fold cost of males.

Given the net two‐fold cost of sex observed here, asexual lineages should rapidly outcompete sexual lineages. Sexual individuals, however, comprised 71.2 ± 1.6% SEM of our field‐collected juveniles from 2012–2015. Previous studies directly demonstrate the long‐term persistence of sexual lineages in *P. antipodarum* (Jokela et al. 2009; Gibson et al. 2016 together span 20 years at Lake Alexandrina). Why do sexual and asexual *P. antipodarum* coexist in nature? One possibility (known as the Red Queen hypothesis) is that coevolving parasites select against common clonal genotypes, thereby giving an advantage to sexual reproduction (Jaenike 1978; Hamilton 1980; Bell 1982; Hamilton et al. 1990). Consistent with this idea, a long‐term field study (Jokela et al. 2009) and a controlled laboratory experiment (Koskella and Lively 2009) both showed that common *P. antipodarum* clones declined in frequency over time after they became disproportionately infected by the sterilizing trematode *Microphallus*. Thus parasite‐mediated frequency‐dependent selection may maintain sexual snail lineages in the face of competition with multiple asexual clones.

What little research there is suggests that the cost of sex varies substantially among systems (Meirmans et al. 2012). Estimating the cost of sex is thus a critical starting point for evaluating the paradox of sex in a natural system. The cost of sex is nonetheless overlooked: hypotheses for the maintenance of sex are often tested in systems for which the cost of sex is unclear. The present study provides a simple framework for estimating the net cost of sex in other species. Our approach has two key requirements. First, it must be possible to separate individuals of different generations (parent vs offspring). Second, the experimental environment must limit selection by extrinsic factors that are known to differentially impact sexual vs. asexual fitness. For example, we made an effort to eliminate selection by coevolving trematode parasites in our seminatural mesocosms. Though we cannot exclude the possibility that our estimate of the net cost of sex in part reflects selection by extrinsic factors, many aspects of the mesocosm environment (e.g., reduced predation, competition) should have reduced differential selection, allowing an estimate of the intrinsic cost of sex.

The long‐term maintenance of sex is one of the core anomalies in evolutionary biology, and the two‐fold cost of sex is the foundational assumption of the paradox. Here, we have provided a straightforward approach to measuring the net cost of sex. Our results provide a quantitative validation of the two‐fold cost of males in a natural system. This large and immediate fitness disadvantage justifies the search for a large and sustained short‐term advantage to cross‐fertilization.

## Supporting information


**Figure S1**. Establishment of experimental mesocosms.
**Figure S2**. Experimental data do not support yearly variation in the cost of sex.

**Table S1**. Numbers of uninfected triploid and diploid parental and offspring snails used in estimating the cost of sex.
**Table S2**. The sex ratio of diploid sexual lineages in the parental generation of experimental mesocosms.
**Table S3**. The sex ratio of diploid sexual lineages in adult snails from field collection sites.
