# Supplementary material for: The two‐fold cost of sex: Experimental evidence from a natural system
Source: Evol Lett. 2017 May 3;1(1):6–15. doi: 10.1002/evl3.1 (PMC6089407; doi:10.1002/evl3.1)
Supplement: Supplementary file 1 — Figure S1. Establishment of experimental mesocosms. Figure S2. Experimental data do not support yearly variation in the cost of sex. [file EVL3-1-6-s001.doc]

**Supporting Information**

The two-fold cost of sex: experimental evidence from a natural system

**Authors:** Amanda K Gibson, Lynda F Delph, and Curtis M Lively

|  |
| --- |
|  |
| **Figure S1: Establishment of experimental mesocosms.** (A) Juvenile snails were collected at ~1 meter depth from well-studied sites along the southwestern coast of Lake Alexandrina (Mackenzie Basin, South Island, New Zealand). In 2012, we sampled the sites Camp and Swamp. In 2013 and 2014, we sampled 1^st^ Fence, Swamp, 2^nd^ Fence, and West Point. In 2015, we sampled Halfway in place of West Point. (B) 800 juveniles were added to ~800 liters of water in Dolav bins outside the University of Canterbury’s Edward Percival Field Station in Kaikoura, New Zealand. The tanks are shown uncovered one year after the start of an experimental run. They are covered with dark shade-cloth during the year. |

**I. Life-history comparisons**

To test the all–else-equal assumption for fecundity, we re-assessed the life-history comparisons of Jokela et al. for field-collected and mesocosm females.

For field comparisons, we collected snails from our study sites at Lake Alexandrina as described in the Methods. When field samples were sieved at 1.7 mm to obtain experimental juvenile snails, we reserved the adult snails (>1.7 mm) for life-history comparison. In 2013- 2015, we determined shell length in millimeters, sex, brood status, and infection status for 150 individuals per site. These data are not available from 2012. For the first 20 females identified as brooding, we counted the number of eggs in the brood. We froze the heads of dissected females and determined reproductive mode for 50 females per site per year using flow cytometry, as outlined in the Methods.

To test if age at reproductive maturity varied with reproductive mode, we fit a linear model with reproductive mode, year, and site as predictors of the length of individual brooding females (Gaussian distribution, identity link function). To test if the probability of brooding varied with reproductive mode, we fit a generalized linear model with brooding status (yes, no) of individual uninfected females as a binomial response variable (logit link function). Females infected with sterilizing trematodes were excluded. Reproductive mode, year and site were categorical factors, and length (proxy for age) was included as a covariate. To test if brood size (a proxy for fecundity) varied with reproductive mode, we fit a generalized linear mode with brood size (negative binomial, log link function) of individual brooding females as the response variable. Predictor variables were identical to those in the analysis of brooding probability. For each model, we tested the significance of reproductive mode using a Wald test. Interactions were tested in each analysis and excluded if insignificant. Analyses were performed in R v3.3.1. We verified that the assumptions of the different models were met.

For mesocosm comparisons, we collected life-history data as described in the main text. For all traits, we fit generalized estimating equations (GEE) with the experimental replicate as the subject variable with an exchangeable variance-covariance matrix. GEEs allow specification of the correlation between individuals derived from the same experimental replicate , and the exchangeable variance-covariance matrix indicates that the correlation between individuals from the same experimental replicate does not vary between replicates . Models were otherwise specified as described for field-collected snails. For variation in length, predictor variables were reproductive mode, year (2012, 2013, 2014, and 2015), and their interaction. For variation in the probability of brooding and in brood size, reproductive mode, year and their interaction were categorical factors, and length (proxy for age) was included as a covariate. Females infected with sterilizing trematodes were excluded from the analysis of brooding probability. The GEE for brood size (negative binomial) was performed in SPSS v23 (IBM). The other GEEs were performed in R. We verified that the assumptions of the different models were met.

In the field, the length of brooding females, a proxy for age at maturity, varied with reproductive mode (n = 106; Wald χ^2^ = 3.9, df = 1, p = 0.05), with asexual females brooding at 96% the length of sexual females, on average (means: asexual – 4.58 mm ± 0.07 SEM vs. sexual – 4.79 ± 0.06). In the mesocosms, we observed no variation with reproductive mode (n = 895; Wald χ^2^ = 3.00, df = 1, p = 0.081; means: asexual – 4.91 mm ± 0.04 vs. sexual – 4.81 ± 0.03). Based upon means and standard errors, sexual females in the mesocosms reached reproductive maturity at the same size as sexual females in the field, while asexual females reached reproductive maturity at a larger size in the mesocosms vs. the field.

In the field, uninfected asexual females were 1.38-fold more likely to be brooding than uninfected sexual females (n = 471; Wald χ^2^ = 7.80, df = 1, p = 0.005; means: asexual – 27.47 ± 2.74% SEM vs. sexual – 19.90 ± 3.38%). In the mesocosms, we observed no variation with reproductive mode (n = 1250; Wald χ^2^ = 0.098, df = 1, p = 0.75; means: asexual – 78.40 ± 3.07% vs. sexual – 71.2 ± 4.35%). Both sexual and asexual females were much more likely to be brooding in the mesocosms than in the field collections. This difference may reflect the distinct age distributions of the field and the mesocosms: the field population includes adults of all ages, from young to old, while the mesocosm populations were composed entirely of young, fertile adults. In addition, the mesocosms excluded many stressors that may reduce investment in reproduction in the field, such as predators, competition (snail densities are likely higher in the field), and disturbance (e.g. wave action).

Lastly, in the field, brooding asexual females carried 55% fewer embryos in their brood pouches than did sexual females (n = 106; Wald χ^2^ = 17.7, df = 1, p<0.001; means: asexual – 9.70 ± 0.93 vs. sexual – 17.51 ± 1.48). In contrast, in the mesocosms, brooding asexual females carried 21% more embryos in their brood pouches than did sexual females (n = 895; Wald χ^2^ = 10.10, df = 1, p = 0.010; means: asexual – 9.65 ± 0.82 vs. sexual – 7.67 ± 0.61). Comparing means and standard errors, brood sizes for sexual females were far lower in the mesocosms than in the field, while brood sizes were similar for asexual females across samples.

As a whole, these life-history comparisons show no clear evidence for a reduction in fecundity associated with the transition to asexual reproduction. In fact, the clonal lineages currently in Lake Alexandrina may have an advantage over sexual females. In mesocosm adults, asexual females had larger brood sizes than sexual females. Consistent with this finding, our estimates of the net cost of sex in the mesocosms suggest that asexual females may have produced a greater number of surviving offspring than sexual females (see Results). In field-collected adults, asexual females had a higher probability of brooding than sexual females, though their broods were approximately half the size of those of sexual females. These findings suggest that field conditions impose additional differential selection upon sexual vs. asexual females. Therefore, the cost of sex realized in the field may differ from the two-fold cost measured in experimental mesocosms.

**II. Experimental test: overdispersion and the quasi-binomial**

For the logistic model used to evaluate differences in the proportion of asexual individuals in parent vs. offspring generations, we tested if the binomial distribution was appropriate for our data using the variance inflation factor (*ĉ)*, which is calculated as the Pearson goodness-of-fit test of model predicted values relative to the model’s residual degrees of freedom. Values for the variance inflation factor that exceed 1 indicate overdispersion . We found evidence that the data were overdispersed with the binomial distribution (*ĉ*=1.93), indicating that the variance in our data exceeded that predicted by the binomial distribution. One way to correct for overdispersion is to correct the estimated variance of model estimates by re-fitting the logistic model with a quasi-binomial distribution . Use of the quasi-binomial does not alter estimates of coefficients, but it can increase the variance of those estimates . Here we compare the results of the binomial model reported in the main text with those of a quasi-binomial model. Comparisons using likelihood ratios are not possible with the quasi-binomial, so we compare estimates of the overall contribution of each predictor using Wald tests.

Use of the quasi-binomial distribution did not qualitatively alter our conclusions. There was an overall effect of generation (binomial: Wald χ^2^ = 25.1, df = 1, p<0.001; quasi-binomial: Wald χ^2^ = 13.0, df = 1, p<0.001), and the frequency of asexual individuals increased substantially from parent to offspring generations (binomial: 1.60, 95% CI [1.48, 1.73]); quasi-binomial: 1.60, [1.43, 1.77]). There was also an effect of year (binomial: Wald χ^2^ = 20.6, df = 3, p<0.001; quasi-binomial: Wald χ^2^ = 10.7, df = 3, p = 0.013), with the overall frequency of asexuals higher in 2013 and 2014 (binomial: odds ratio vs. 2012: 2013, 1.70 [1.39, 2.07], 2014, 1.43 [1.17, 1.75], 2015, 1.15 [0.94, 1.41]; quasi-binomial: odds ratio vs. 2012: 2013, 1.70 [1.30, 2.22], 2014, 1.43 [1.09, 1.88], 2015, 1.15 [0.87, 1.51]). There was no overall effect of the interaction of generation and year (binomial: Wald χ^2^ = 2.4, df = 3, p = 0.500; quasi-binomial: Wald χ^2^ = 1.2, df = 3, p = 0.75).

**III. Model fit: selection of the beta-binomial distribution**

To apply equation (2) to our mesocosm data, we initially assumed a binomial distribution for the likelihood functions and found that the variance inflation factor for our global model (candidate model 4) was substantially greater than 1 (ĉ=4.08). A variance inflation factor of this magnitude is consistent with severe overdispersion and indicates that the binomial distribution is not appropriate for our data. We therefore assumed a beta-binomial distribution, which better models variation by estimating an additional parameter *θ* to allow for variation in the per-trial probability . Small estimates of *θ* are consistent with larger overdispersion . A likelihood ratio test comparing the global model with a binomial vs. beta-binomial distribution strongly justified use of the beta-binomial (χ^2^ = 46.67, df = 1, p<0.001). Similarly, the beta-binomial model had a lower AIC_c_, and ΔAIC_c_ was very large (43.45). Consistent with these results, the maximum likelihood estimate of *θ* was small (20.23) (global model).

**IV. Tertiary sex ratio in field-collected parents and mesocosm adults**

To estimate a range for the primary sex ratio (*s*), we examined the tertiary sex ratio of *P. antipodarum*, which we calculated as the proportion of adult sexual females in the adult sexual subpopulations of our study populations. This proportion was calculated similarly to the proportion of asexual females, but infected individuals were included in the calculations (Tables S2 and S3). For mesocosm parents, we fit a binomial distribution to the number of diploid female and male snails in our 24 experimental replicates (Table S2) and used the mle2 function (package bbmle, R) to find the maximum likelihood estimate of the probability that a diploid snail is female (sex ratio). We used the function confint to obtain 95% confidence intervals on the estimate. We did the same for field-collected adults, fitting a binomial distribution to the number of diploid female and male snails at our sampled field sites in 2013-2015 (Table S3). We used a likelihood ratio test to compare these models against models in which the binomial probability was fixed at 0.5.

For mesocosm parents, the maximum likelihood estimate of the tertiary sex ratio was 61% female (95% CI [0.59, 0.64]). For field-collected adults, the maximum likelihood estimate of the tertiary sex ratio was 70% female (95% CI [0.66, 0.74]). For both samples, these models fit our data substantially better than a model with the sex ratio fixed at 50% female (likelihood ratio test: mesocosms - D = 71.22, df = 1, p<0.001; field – D = 79.65, df = 1, p<0.001). The tertiary sex ratio was more female-biased in the field’s sexual population than in the mesocosms’. Early male mortality may explain the female bias in tertiary sex ratios and could additionally contribute to the more extreme bias in the field population. Based upon these results, we concluded that the primary sex ratio likely lies between 50% and 61% female (0.5 ≤ *s* ≤ 0.61).

**V. Model fit: yearly variation in the cost of sex**

As discussed in the main text, there was weak support for candidate model 4, in which the magnitude of the cost of sex varies between the four years in which the experiment was replicated (ΔAIC_c_ = 8.93, *w* = 0.01). Parameter estimates from model 4 are consistent with this finding. The maximum likelihood estimate of the baseline cost in 2012 was slightly below two (*c_0_* = 1.92, 95% CI [1.34, 2.59]). The maximum likelihood estimate for the difference from this 2012 baseline included zero for 2013 (*d_2_* = 0.19, [-0.62, 1.01]), 2014 (*d_3_* = 0.85, [-0.03, 1.78]), and 2015 (*d_4_* = 0.007, [-0.85, 0.91]), indicating no significant difference from 2012 (Fig. S2).

| 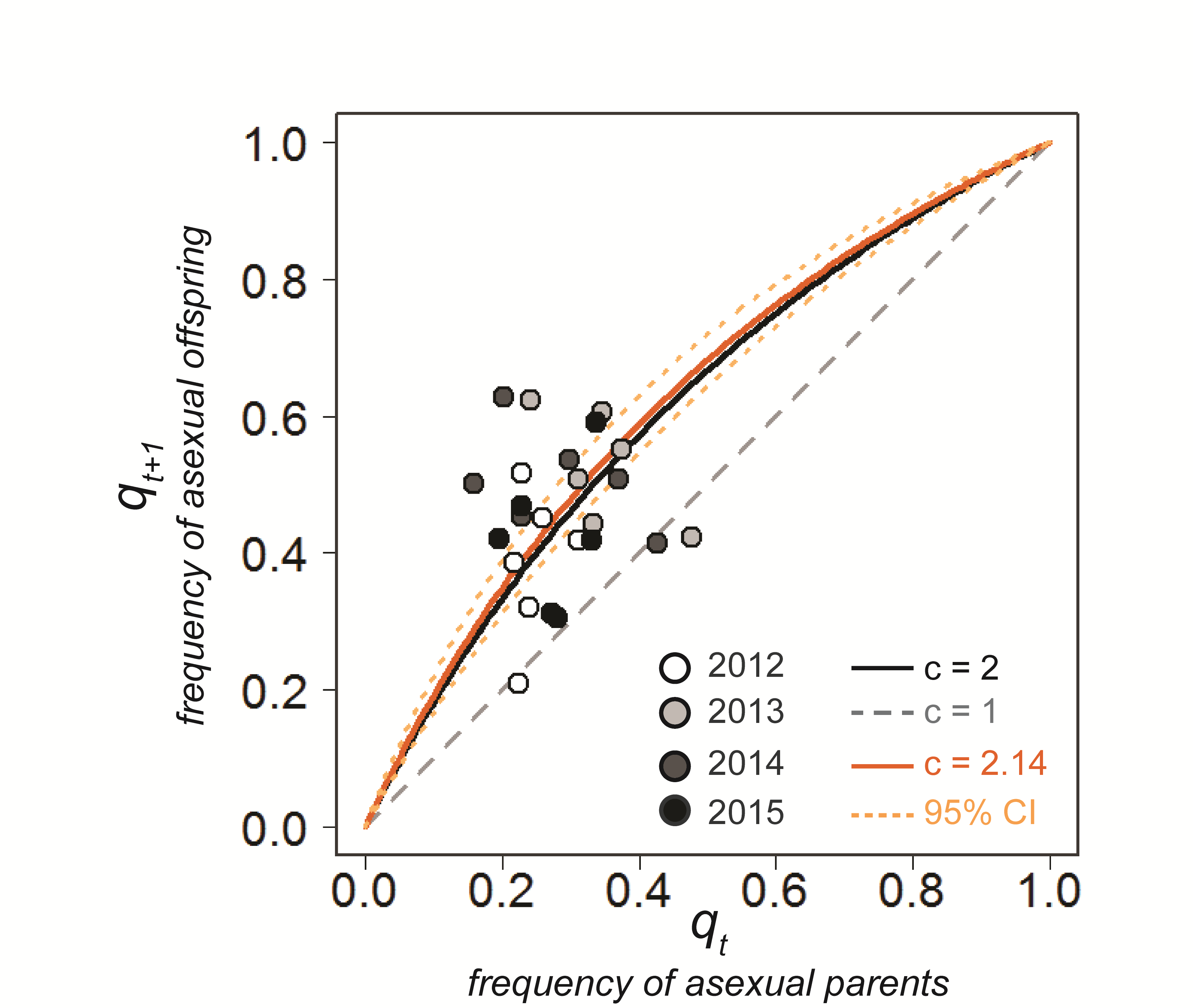 |
| --- |
| **Figure S2: Experimental data do not support yearly variation in the cost of sex.** As in Figure 3, we fit our theoretical formulation of the cost of sex (Fig. 2c; equation (2)) to experimental data (Fig. 1b) on the frequency of asexuals *q* in generations *t* and *t+1* in 24 semi-natural mesocosms (points). The predicted frequency of asexual offspring (*q_t+1_*) for a given frequency of asexual parents (*q_t_*) is shown for three values of the cost of sex: no cost (*c*=1, gray dashed line), a two-fold cost (c=2, black solid line), and the maximum likelihood estimate (*c*=2.14, solid orange line; 95% confidence intervals = [1.81, 2.55], dotted orange lines). Each point represents one mesocosm, with points colored according to year: 2012 (white), 2013 (light gray), 2014 (dark gray), and 2015 (black). We found no evidence for significantly yearly variation in the cost of sex: the estimate of *c* in 2012 was lower than that in 2013-2015, but not significantly so. |

**References**

Bolker, B. 2008. Ecological Models and Data in R. Princeton University Press.

Burnham, K. P. and D. R. Anderson. 1998. Model Selection and Inference: a Practical Information-Theoretic Approach. Springer, New York.

Cox, D. R. and E. J. Snell. 1989. Analysis of binary data. CRC, Boca Raton, FL.

Crawley, M. J. 2013. Chapter 16: Proportion Data. The R Book. John Wiley & Sons, Ltd. , West Sussex, UK.

Crowder, M. 1978. Beta-binomial ANOVA for proportions. J Roy Stat Soc C 27:34-37.

Jokela, J., C. M. Lively, M. F. Dybdahl, and J. Fox. 1997. Evidence for a cost of sex in the freshwater snail *Potamopyrgus antipodarum*. Ecology 78:452-460.

Liang, K.-Y. and S. Zeger. 1986. Longitudinal data analysis using generalized linear models. Biometrika 73:13-22.

Venables, W. and B. Ripley. 2002. Modern Applied Statistics with S. Springer, New York City, NY.

Zeger, S. and K.-Y. Liang. 1986. Longitudinal data analysis for discrete and continuous outcomes. Biometrics 42:121-130.

Zuur, A., E. Ieno, N. Walker, A. Saveliev, and G. Smith. 2009. Generalised Estimating Equations *in* A. Zuur, E. Ieno, N. Walker, A. Saveliev, and G. Smith, eds. Mixed Effects Models and Extensions in Ecology with R. Springer.
