# Supplementary material for: The two‐fold cost of sex: Experimental evidence from a natural system
Source: Evol Lett. 2017 May 3;1(1):6–15. doi: 10.1002/evl3.1 (PMC6089407; doi:10.1002/evl3.1)
Supplement: Supplementary file 2 — Table S1. Numbers of uninfected triploid and diploid parental and offspring snails used in estimating the cost of sex. Table S2. The sex ratio of diploid sexual lineages in the parental generation of experimental mesocosms. Table S3. The sex ratio of diploid sexual lineages in adult snails from field collection sites. [file EVL3-1-6-s002.doc]

| **Table S1: Numbers of uninfected triploid and diploid parental and offspring snails used in estimating the cost of sex.** For the parental generation, we dissected a large, random sample of snails from each mesocosm to determine sex. Males were assumed to be diploid sexuals. We analyzed the reproductive mode of a random subsample of females. The number of triploid and diploid females in the subsample were obtained directly from these flow cytometry results. To determine the total number of snails that were diploid (sexual males and females) vs. triploid (asexual females), we used the ratio of male to female snails in the larger mesocosm sample to calculate the number of males corresponding to the subsample of females analyzed with flow cytometry. Infected individuals were excluded from these calculations. For the offspring generation, we analyzed the reproductive mode of all snails, male and female, using flow cytometry, because the offspring snails were too young to sex. We therefore determine the number of diploid vs. triploid offspring directly from the flow cytometry results. |
| --- |
| |  |  |  | **Dissected sample** | | | **Flow cytometry subsample** | | | **Estimates** | | | | | --- | --- | --- | --- | --- | --- | --- | --- | --- | --- | --- | --- | --- | | Year | Mesocosm | Generation | N ♀ | N ♂ | Total | N diploid ♀ | N triploid ♀ | Total | Corresponding N ♂ | N diploid ♀, ♂ | Total diploid ♀, ♂ and triploid ♀ | % triploid | | 2012 | 1 | parent | 62 | 35 | 97 | 22 | 21 | 43 | 24 | 46 | 67 | 31.22 | | 2012 | 2 | parent | 90 | 48 | 138 | 55 | 30 | 85 | 45 | 100 | 130 | 23.02 | | 2012 | 3 | parent | 113 | 29 | 142 | 62 | 30 | 92 | 24 | 86 | 116 | 25.95 | | 2012 | 4 | parent | 97 | 54 | 151 | 60 | 31 | 91 | 51 | 111 | 142 | 21.88 | | 2012 | 5 | parent | 103 | 37 | 140 | 60 | 29 | 89 | 32 | 92 | 121 | 23.97 | | 2012 | 6 | parent | 91 | 45 | 136 | 57 | 29 | 86 | 43 | 100 | 129 | 22.56 | | 2013 | 1 | parent | 95 | 40 | 135 | 25 | 20 | 45 | 19 | 44 | 64 | 31.28 | | 2013 | 2 | parent | 84 | 40 | 124 | 25 | 14 | 39 | 19 | 44 | 58 | 24.32 | | 2013 | 3 | parent | 93 | 36 | 129 | 21 | 18 | 39 | 15 | 36 | 54 | 33.27 | | 2013 | 4 | parent | 87 | 43 | 130 | 18 | 23 | 41 | 20 | 38 | 61 | 37.54 | | 2013 | 5 | parent | 93 | 44 | 137 | 22 | 23 | 45 | 21 | 43 | 66 | 34.70 | | 2013 | 6 | parent | 94 | 34 | 128 | 15 | 28 | 43 | 16 | 31 | 59 | 47.82 | | 2014 | 1 | parent | 106 | 30 | 136 | 22 | 20 | 42 | 12 | 34 | 54 | 37.12 | | 2014 | 2 | parent | 103 | 32 | 135 | 28 | 12 | 40 | 12 | 40 | 52 | 22.89 | | 2014 | 3 | parent | 96 | 36 | 132 | 19 | 27 | 46 | 17 | 36 | 63 | 42.69 | | 2014 | 4 | parent | 98 | 36 | 134 | 29 | 20 | 49 | 18 | 47 | 67 | 29.85 | | 2014 | 5 | parent | 91 | 35 | 126 | 28 | 11 | 39 | 15 | 43 | 54 | 20.37 | | 2014 | 6 | parent | 101 | 39 | 140 | 35 | 10 | 45 | 17 | 52 | 62 | 16.03 | | 2015 | 1 | parent | 78 | 49 | 127 | 18 | 21 | 39 | 25 | 43 | 64 | 33.07 | | 2015 | 2 | parent | 97 | 44 | 141 | 26 | 17 | 43 | 20 | 46 | 63 | 27.20 | | 2015 | 3 | parent | 96 | 40 | 136 | 29 | 14 | 43 | 18 | 47 | 61 | 22.98 | | 2015 | 4 | parent | 90 | 42 | 132 | 27 | 11 | 38 | 18 | 45 | 56 | 19.74 | | 2015 | 5 | parent | 109 | 31 | 140 | 30 | 17 | 47 | 13 | 43 | 60 | 28.16 | | 2015 | 6 | parent | 102 | 42 | 144 | 22 | 20 | 42 | 17 | 39 | 59 | 33.73 | | 2012 | 1 | offspring |  |  |  |  | 23 |  |  | 32 |  | 41.82 | | 2012 | 2 | offspring |  |  |  |  | 30 |  |  | 28 |  | 51.72 | | 2012 | 3 | offspring |  |  |  |  | 37 |  |  | 45 |  | 45.12 | | 2012 | 4 | offspring |  |  |  |  | 22 |  |  | 35 |  | 38.60 | | 2012 | 5 | offspring |  |  |  |  | 18 |  |  | 38 |  | 32.14 | | 2012 | 6 | offspring |  |  |  |  | 12 |  |  | 45 |  | 21.05 | | 2013 | 1 | offspring |  |  |  |  | 37 |  |  | 36 |  | 50.68 | | 2013 | 2 | offspring |  |  |  |  | 43 |  |  | 26 |  | 62.32 | | 2013 | 3 | offspring |  |  |  |  | 31 |  |  | 39 |  | 44.29 | | 2013 | 4 | offspring |  |  |  |  | 38 |  |  | 31 |  | 55.07 | | 2013 | 5 | offspring |  |  |  |  | 43 |  |  | 28 |  | 60.56 | | 2013 | 6 | offspring |  |  |  |  | 30 |  |  | 41 |  | 42.25 | | 2014 | 1 | offspring |  |  |  |  | 35 |  |  | 34 |  | 50.72 | | 2014 | 2 | offspring |  |  |  |  | 29 |  |  | 35 |  | 45.31 | | 2014 | 3 | offspring |  |  |  |  | 29 |  |  | 41 |  | 41.43 | | 2014 | 4 | offspring |  |  |  |  | 37 |  |  | 32 |  | 53.62 | | 2014 | 5 | offspring |  |  |  |  | 42 |  |  | 25 |  | 62.69 | | 2014 | 6 | offspring |  |  |  |  | 34 |  |  | 34 |  | 50.00 | | 2015 | 1 | offspring |  |  |  |  | 28 |  |  | 39 |  | 41.79 | | 2015 | 2 | offspring |  |  |  |  | 20 |  |  | 44 |  | 31.25 | | 2015 | 3 | offspring |  |  |  |  | 30 |  |  | 34 |  | 46.88 | | 2015 | 4 | offspring |  |  |  |  | 29 |  |  | 40 |  | 42.03 | | 2015 | 5 | offspring |  |  |  |  | 21 |  |  | 48 |  | 30.43 | | 2015 | 6 | offspring |  |  |  |  | 39 |  |  | 27 |  | 59.09 | |

| **Table S2: The sex ratio of diploid sexual lineages in the parental generation of experimental mesocosms.** Infected individuals were included in these calculations. For the parental generation, we dissected a large, random sample of snails from each mesocosm to determine sex. Males were assumed to be diploid sexuals. We analyzed the reproductive mode of a random subsample of females. The number of diploid females in the subsample were obtained directly from these flow cytometry results. We then used the ratio of male to female snails in the larger mesocosm sample to calculate the number of males corresponding to the subsample of females analyzed with flow cytometry. We then calculated the proportion of diploid (sexual) individuals that were female as an estimate of *s,* the primary sex ratio. |
| --- |
| |  |  |  | **Dissected sample** | | | **Flow cytometry subsample** | | | **Estimates** | | |  | | | --- | --- | --- | --- | --- | --- | --- | --- | --- | --- | --- | --- | --- | --- | | Year | Mesocosm | Generation | N ♀ | N ♂ | Total | N diploid ♀ | N triploid ♀ | Total | Corresponding N ♂ | N diploid ♀, ♂ | Sex ratio: proportion diploids that are ♀ | |  | | 2012 | 1 | parent | 66 | 35 | 101 | 23 | 23 | 46 | 24 | 47 | 48.53 | |  | | 2012 | 2 | parent | 99 | 51 | 150 | 61 | 33 | 94 | 48 | 109 | 55.75 | |  | | 2012 | 3 | parent | 121 | 29 | 150 | 67 | 31 | 98 | 23 | 90 | 74.04 | |  | | 2012 | 4 | parent | 105 | 54 | 159 | 66 | 33 | 99 | 51 | 117 | 56.45 | |  | | 2012 | 5 | parent | 115 | 37 | 152 | 70 | 30 | 100 | 32 | 102 | 68.51 | |  | | 2012 | 6 | parent | 103 | 48 | 151 | 65 | 32 | 97 | 45 | 110 | 58.98 | |  | | 2013 | 1 | parent | 107 | 43 | 150 | 26 | 22 | 48 | 19 | 45 | 57.41 | |  | | 2013 | 2 | parent | 104 | 46 | 150 | 30 | 19 | 49 | 22 | 52 | 58.06 | |  | | 2013 | 3 | parent | 110 | 40 | 150 | 29 | 21 | 50 | 18 | 47 | 61.46 | |  | | 2013 | 4 | parent | 105 | 45 | 150 | 23 | 26 | 49 | 21 | 44 | 52.27 | |  | | 2013 | 5 | parent | 105 | 45 | 150 | 27 | 23 | 50 | 21 | 48 | 55.75 | |  | | 2013 | 6 | parent | 116 | 34 | 150 | 18 | 31 | 49 | 14 | 32 | 55.62 | |  | | 2014 | 1 | parent | 118 | 32 | 150 | 26 | 21 | 47 | 13 | 39 | 67.10 | |  | | 2014 | 2 | parent | 115 | 35 | 150 | 33 | 13 | 46 | 14 | 47 | 70.21 | |  | | 2014 | 3 | parent | 109 | 41 | 150 | 23 | 27 | 50 | 19 | 42 | 55.01 | |  | | 2014 | 4 | parent | 112 | 38 | 150 | 33 | 23 | 56 | 19 | 52 | 63.46 | |  | | 2014 | 5 | parent | 113 | 37 | 150 | 33 | 13 | 46 | 15 | 48 | 68.66 | |  | | 2014 | 6 | parent | 109 | 41 | 150 | 37 | 11 | 48 | 18 | 55 | 67.21 | |  | | 2015 | 1 | parent | 98 | 52 | 150 | 23 | 25 | 48 | 25 | 48 | 47.45 | |  | | 2015 | 2 | parent | 106 | 44 | 150 | 29 | 17 | 46 | 19 | 48 | 60.30 | |  | | 2015 | 3 | parent | 110 | 40 | 150 | 33 | 16 | 49 | 18 | 51 | 64.94 | |  | | 2015 | 4 | parent | 106 | 43 | 149 | 33 | 11 | 44 | 18 | 51 | 64.90 | |  | | 2015 | 5 | parent | 114 | 36 | 150 | 32 | 17 | 49 | 15 | 47 | 67.41 | |  | | 2015 | 6 | parent | 108 | 42 | 150 | 25 | 21 | 46 | 18 | 43 | 58.29 | |  | |

| **Table S3: The sex ratio of diploid sexual lineages in adult snails from field collection sites.** Infected individuals were included in these calculations. We dissected a large, random sample of adult snails from each field site in 2013-2015 to determine sex. Males were assumed to be diploid sexuals. We analyzed the reproductive mode of a random subsample of females. To estimate *s,* the primary sex ratio, we obtained the proportion of diploid (sexual) individuals that were female as described in Table S2. |
| --- |
| |  |  | **Dissected sample** | | | **Flow cytometry subsample** | | | **Estimates** | | |  | | | --- | --- | --- | --- | --- | --- | --- | --- | --- | --- | --- | --- | --- | | Year | Site | N ♀ | N ♂ | Total | N diploid ♀ | N triploid ♀ | Total | Corresponding N ♂ | N diploid ♀, ♂ | Sex ratio: proportion diploids that are ♀ | |  | | 2013 | 1^st^ Fence | 115 | 35 | 150 | 20 | 12 | 32 | 10 | 30 | 67.25 | |  | | 2013 | Swamp | 116 | 34 | 150 | 23 | 25 | 48 | 14 | 37 | 62.05 | |  | | 2013 | 2^nd^ Fence | 122 | 28 | 150 | 26 | 13 | 39 | 9 | 35 | 74.39 | |  | | 2013 | West Point | 121 | 29 | 150 | 27 | 22 | 49 | 12 | 39 | 69.69 | |  | | 2014 | 1^st^ Fence | 107 | 43 | 150 | 32 | 14 | 46 | 18 | 50 | 63.38 | |  | | 2014 | Swamp | 119 | 31 | 150 | 29 | 21 | 50 | 13 | 42 | 69.01 | |  | | 2014 | 2^nd^ Fence | 116 | 34 | 150 | 35 | 16 | 51 | 15 | 50 | 70.07 | |  | | 2014 | West Point | 130 | 20 | 150 | 20 | 28 | 48 | 7 | 27 | 73.03 | |  | | 2015 | 1^st^ Fence | 112 | 38 | 150 | 38 | 10 | 48 | 16 | 54 | 70.00 | |  | | 2015 | Swamp | 127 | 23 | 150 | 28 | 18 | 46 | 8 | 36 | 77.07 | |  | | 2015 | 2^nd^ Fence | 120 | 30 | 150 | 43 | 17 | 60 | 15 | 58 | 74.14 | |  | | 2015 | Halfway | 131 | 19 | 150 | 16 | 31 | 47 | 7 | 23 | 70.12 | |  | |
